# Combination of Slightly Acidic Electrolyzed Water and Hydrogel to Enhance Stability, Increase Antibacterial Efficacy, and Promote Infectious Wound Healing

**DOI:** 10.3390/ijms26125908

**Published:** 2025-06-19

**Authors:** Nanxin Li, Chao Li, Dongbo Li, Awn Abbas, Xingyu Chen, Xiaoyang Ai, Wei Zhang, Gang Shu, Juchun Lin, Haohuan Li, Funeng Xu, Guangneng Peng, Hualin Fu

**Affiliations:** Department of Pharmacy, College of Veterinary Medicine, Sichuan Agricultural University, Chengdu 611130, China; linanxinao@163.com (N.L.); 15908499457@163.com (C.L.); 18081626755@163.com (D.L.); awni1115@gmail.com (A.A.); 13857673234@163.com (X.C.); a1600063002@163.com (X.A.); zhangwei26510c@126.com (W.Z.); dyysg2005@sicau.edu.cn (G.S.); juchunlin@126.com (J.L.); lihaohuan7@163.com (H.L.); funengxu@sicau.edu.cn (F.X.); pgn.sicau@163.com (G.P.)

**Keywords:** slightly acidic electrolyzed water, hydrogel, stability, antibacterial activity, wound healing

## Abstract

Wound infections remain significant challenges for current tissue adhesives, primarily due to their poor adhesion in moist environments, slow bonding, cytotoxicity, and limited antibacterial properties. Slightly acidic electrolyzed water (SAEW), a potent disinfectant, suffers from limited stability due to chlorine loss. This study developed a novel SAEW-based hydrogel (SAEW-gel) by combining SAEW with chitosan and β-glycerol disodium phosphate to improve its stability and therapeutic potential. SAEW-gel demonstrated high water absorption, long-term water retention, and enhanced antibacterial activity against *S. aureus* and *E. coli* compared to SAEW alone. It maintained germicidal efficacy after prolonged storage and significantly accelerated wound healing in a rat model, achieving a 95.41% healing rate by the 12th day of treatment. Mechanistically, SAEW-gel reduced inflammatory cell infiltration, promoted granulation and collagen formation, and regulated inflammatory markers (IL-6, IL-1β, TNF-α, MPO, HYP). These findings highlight SAEW-gel as a promising biomaterial for treating infectious wounds and support its potential for future clinical application.

## 1. Introduction

Wound infections represent a significant global healthcare burden, particularly in patients with chronic wounds or severe trauma. These infections not only delay the healing process but can also lead to serious complications such as sepsis, organ dysfunction, and even death. Recent studies have reported that over 60% of patients with severe traumatic wounds develop infections, contributing to high rates of respiratory distress and multiorgan failure, with associated mortality rates reaching up to 27%. Wound healing is a complex and multiphase process. Prolonged inflammation is a major cause of excessive scar formation and is considered the main barrier in the healing of chronic wounds [1]. Chronic wounds such as diabetic foot ulcers (DFUs) are mostly caused by immune dysregulation and excessive inflammation. In DFUs, macrophages do not shift from a pro-inflammatory (M1) to a reparative (M2) phase as they normally do during the healing process. This persistent M1 polarization inhibits tissue regeneration, delays the healing process, and increases the risk of infection, which potentially lead to some serious complications [2]. The early detection and management of wound infections are crucial to promote healing, reduce hospital stays, and lower healthcare expenditures. However, conventional diagnostic methods are often invasive, subjective, and incapable of accurately assessing infection status in a timely manner [3]. Given the severity and complexity of wound infections, there is a pressing need to develop novel therapeutic strategies. Advanced topical treatments, such as antimicrobial hydrogels, have emerged as promising alternatives due to their ability to disrupt biofilms and minimize antibiotic resistance, ultimately improving healing outcomes and patient quality of life.

Electrolyzed water, a groundbreaking innovation in green chemistry, has emerged as a novel disinfectant with extensive applications across various domains, including disinfection [4,5], food hygiene [6,7], etc. SAEW, characterized by an available chlorine concentration (ACC) ranging from 10 to 60 mg/L and a pH of 5.0 to 6.5, is produced through the electrolysis of a dilute HCl solution in a nonmembrane electrolysis apparatus [8]. Previous studies [9,10] have demonstrated the effectiveness of SAEW in eliminating a broad range of microorganisms, including *Escherichia coli*, *Staphylococcus aureus*, *Pseudomonas aeruginosa*, and the spores of *Bacillus subtilis* var. The primary active chlorine compound in SAEW is hypochlorous acid (HClO), which exhibits significantly higher bactericidal activity against *Escherichia coli* compared to a considerable concentration of hypochlorite ions (ClO^−^), with an efficacy 80 times greater over the same treatment duration [11,12]. Compared with the use of SAEW (pH < 2.5), the application of SAEW can reduce the adverse effects of chlorine emissions on human health, the environment, and equipment corrosion. Despite its benefits, SAEW’s efficacy is limited by its heavy reliance on equipment and the significant chlorine loss. Chlorine loss in SAEW is primarily influenced by external factors, particularly exposure to air and light [13]. Wang [14] discovered that storing SAEW in a closed environment at 4 °C (resulting in approximately a 30% decrease in ACC) is more effective in maintaining its stability compared to open storage at 20 °C. Len [15] also reported that under open conditions, the loss of chlorine in electrolyzed water is primarily due to the evaporation of chlorine into the atmosphere. Consequently, minimizing SAEW’s exposure to the external environment can effectively enhance its stability.

Hydrogels constitute an emerging class of biomaterial dressings with the capacity to efficiently encapsulate and deliver therapeutic agents. Their loose, porous structure grants superior permeability to low-molecular-weight solutes, and their ability to be modified allows them to adapt to various physiological environments within the body. Hydrogels can modulate drug release through their swelling behavior and intelligent responsiveness to environmental stimuli, functioning as an effective drug delivery system that maintains a stable drug concentration over extended periods [16]. Additionally, hydrogels possess good water absorption and retention properties, enabling them to absorb and sustain the release of significant amounts of water [17]. Chitosan, derived from the deacetylation of chitin, is a naturally occurring linear polysaccharide composed of amino sugar units. It is formed by the linkage of glucosamine and *N*-acetylglucosamine units through β-(1–4) glycosidic bonds [18]. Chitosan, soluble in acidic environments, is unique among natural polysaccharides for its positively charged surface. Its abundant amino and hydroxyl groups, along with partial amide linkages, facilitate the formation of hydrogen bonds between molecules. Chitosan boasts excellent biocompatibility, biodegradability, and antibacterial properties, as well as low toxicity [19]. In recent years, it has emerged as a popular and readily preparable substrate for novel antibacterial hydrogels [20]. Chitosan hydrogels not only exhibit excellent water absorption and retention properties, as well as effective sustained drug release capabilities, but also demonstrate strong antibacterial activity. Current studies have shown that chitosan-based hydrogels possess good antimicrobial capabilities. Zhao et al. [21] demonstrated that chitosan nanoparticle composites show strong bactericidal activity against *S. aureus* and *E. coli*, while chitosan–hyaluronic acid hydrogels effectively inhibit biofilm formation and support tissue regeneration [22]. Peers [16] highlighted that chitosan gel exhibits an excellent sustained and controlled release effect on both hydrophilic and hydrophobic drugs, attributable to its intriguing properties, including its biocompatibility, biodegradability, antibacterial activity, and adhesive characteristics. These properties of the gel enable it to extend the duration of drug action effectively. Harrar [23] synthesized an innovative hydrogel nanocomposite (Cs-g-PAAM/Ag NPS) by incorporating chitosan-grafted polyacrylamide with silver nanoparticles. This composite material exhibited a robust antibacterial effect against *Staphylococcus aureus* and *Escherichia coli*, reaching a maximum bactericidal rate of 99.99%. This outcome underscores the excellent antibacterial properties inherent in chitosan hydrogels.

This study aims to develop a hydrogel based on chitosan that can effectively encapsulate a significant amount of SAEW by using the unique properties of chitosan, which include high water absorption, superior water retention, and strong sustained release. This hydrogel-based approach also aims to increase antibacterial activity by maintaining an enhanced release time and improved stability properties. Our developed dressing has multipurpose potential, such as promoting wound healing and infection control in emergency wound care, particularly in situations where traditional closure methods are inadequate.

## 2. Results and Discussion

### 2.1. Properties of SAEW and SAEW-Gel

**Figure 1:**
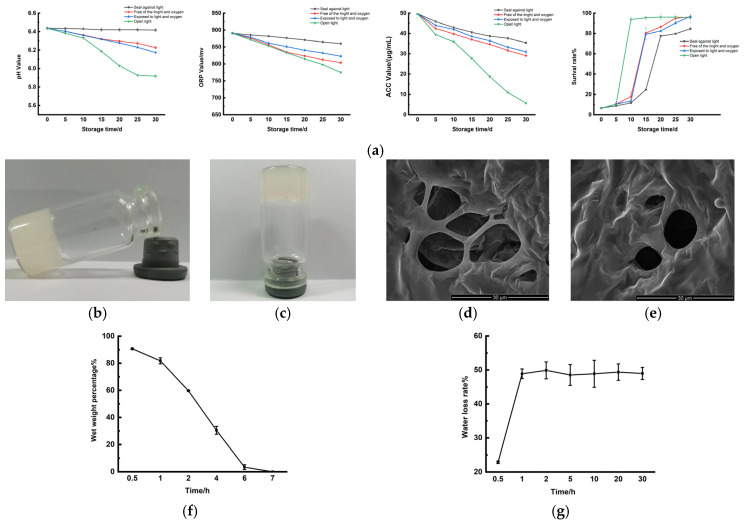
Properties of SAEW and SAEW-gel. (**a**) Stability of SAEW. (**b**) SAEW-gel tilted for 5 min. (**c**) SAEW-gel inverted for 5 min. (**d**) SEM image of CS-gel; (**e**) SEM image of SAEW-gel. (**f**) Dissolution properties of SAEW-gel. (**g**) Water retention properties of SAEW-gel.

Under sealed conditions to prevent light exposure, the pH of SAEW remains relatively constant over 30 days, whereas when opened and exposed to light storage, the pH of SAEW exhibits the most significant decrease within 30 days, decreasing by approximately 0.52 (Figure 1a). In open and exposed conditions, two reactions occur simultaneously, leading to a substantial pH drop. In contrast, under open but shaded conditions and sealed storage, the pH decreases by about 0.21 and 0.26, respectively, over 30 days. The difference between these two conditions is minimal, at 0.05, indicating that the effects of light exposure and air exposure on the pH of SAEW are similar. Under the four storage conditions of open with light, open without light, sealed with light, and sealed without light, the ORP and ACC values of the SAEW showed a degree of decrease over 30 days. The specific changes in ORP and ACC values are as follows: open with light (about 116 mv, 13.03%) > open without light (about 86 mv, 9.66%) > sealed with light (about 68 mv, 7.64%) > sealed without light (about 30 mv, 37%). The values of ACC decrease as follows: open with light (about 44.03 μg/mL, 88.52%) > open without light (20.68 μg/ mL, 41.57%) > sealed with light (18.77 μg/mL, 37.73%) > sealed without light (14.30 μg/mL, 28.74%). The concentration of ACC in SAEW is a pivotal factor influencing its antibacterial efficacy. The antibacterial activity of SAEW against *S. aureus* over the 30-day observation period exhibited a gradual decline. When exposed to light, SAEW’s antimicrobial capacity experienced a marked reduction around day 10. In contrast, under conditions of darkness while being open and light exposure in a closed environment, a decline in antibacterial activity was observed around day 15. For SAEW that was sealed and shielded from light, a significant decrease in antibacterial capability was noted around the 20th day. SAEW is highly unstable, primarily due to its significant chlorine loss. It typically degrades into ordinary water over time when exposed to light and air. Two primary factors contribute to the decrease in pH of SAEW [11]. First, the gradual dissolution of atmospheric CO_2_ in SAEW leads to the formation of carbonic acid (H_2_CO_3_), which lowers the pH. Second, the hypochlorous acid (HClO) present in SAEW undergoes rapid decomposition into H^+^, Cl^−^, and ClO^−^ when exposed to light, thereby further facilitating a reduction in pH. Under the condition of open storage, the ORP value of SAEW remains relatively stable. This stability is attributed to the fact that, while HClO may volatilize after decomposition under open conditions, the dissolved CO_2_ in the air can replenish some oxidized substances in the slightly acidic electrolytic water, resulting in only a minimal overall change in the ORP value. Over the 30-day storage period, it was observed that the available chlorine in the SAEW experienced varying degrees of depletion, regardless of the storage conditions. This depletion accelerated following exposure to light or air. During its interaction with air, SAEW absorbs CO_2_, resulting in relatively stable pH and ORP values, although both parameters exhibit a downward trend over time. The recommended optimal storage conditions for SAEW are a dark, sealed environment to minimize these effects.

As observed, SAEW-gel exhibits an opaque gel-like consistency (Figure 1b), aiding in the preservation of SAEW. After being tilted and inverted for 5 min, the morphology of the SAEW-gel remained unchanged (Figure 1c). Both the chitosan hydrogel (Figure 1d) and the SAEW-gel (Figure 1e) display a characteristic loose and porous network structure, with no significant differences between them. SAEW-gel dissolves almost entirely (3.41%) within 6 h (Figure 1f), demonstrating effective dissolution characteristics. The water loss rate of SAEW-gel is notably low at 48.97% and remains stable over 30 days (Figure 1g). This certainly proves its improved water retention and stability over a longer period as compared to SAEW alone.

Building upon previous research, it is understood that the thermosensitive nature of the hydrogel arises from chitosan molecules forming hydrogen bonds with water molecules at lower temperatures. This prevents the formation of hydrogen bonds between the free amino and hydroxyl groups within the chitosan molecular chain, allowing for a regular arrangement of the chitosan molecules and avoiding entanglement. However, as temperature increases, the hydrogen bonds between chitosan and water molecules are disrupted and the electrostatic forces between chitosan molecules are altered due to the addition of β-glycerophosphate disodium hydrate. The attraction between the hydrophobic and hydrogen-bonding regions of chitosan becomes stronger than the electrostatic forces between the molecular bonds, leading to some segments physically binding to the chitosan molecular bonds and the solution condensing to form a hydrogel [24].

Throughout the 90-day storage period, SAEW-gel demonstrated robust antibacterial properties and stability, effectively addressing the issue of SAEW’s inherent instability. This improvement is speculated to be closely associated with the inherent properties of chitosan hydrogels. These findings conclusively illustrate that SAEW-gel can significantly prolong the service life of SAEW and enhance its overall stability.

### 2.2. Evaluation of Antibacterial Activity In Vitro

**Figure 2:**
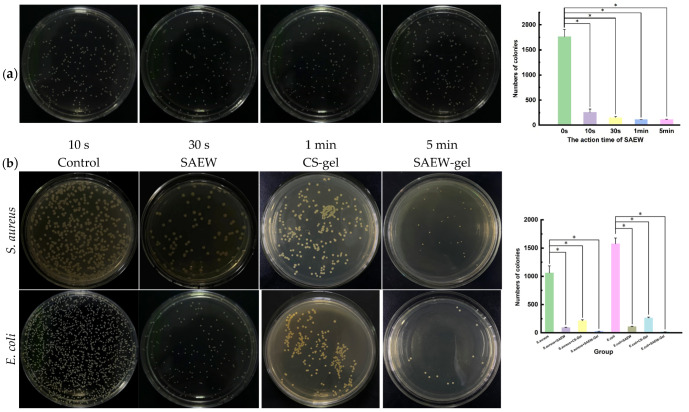
Antibacterial activity of SAEW and SAEW-gel. (**a**) SAEW reactions with *Escherichia coli* over different times. (**b**) Antibacterial activity of SAEW and SAEW-gel against *S. aureus* and *E. coli*. The asterisk (*) indicates a statistically significant difference (* *p* < 0.05).

We investigated the antibacterial effects of SAEW on *Escherichia coli* over varying contact times (Figure 2a). Notably, SAEW exhibits a significant antibacterial impact within just 10 s of exposure to *E. coli*, with the efficacy approaching its maximum within 1 min. Consequently, for subsequent antibacterial experiments, the exposure time for SAEW was standardized at 1 min to ensure optimal antibacterial activity.

The survival rates of *S. aureus* and *E. coli* treated with SAEW for 1 min were significantly reduced to 6.61% and 5.32% (Figure 2b), respectively, demonstrating potent antibacterial activity (*p* < 0.05). This bactericidal mechanism is attributed to hypochlorous acid, which can damage the cell membrane, leading to the disruption of the normal biochemical activities of proteases, RNA, and DNA within the cells, ultimately resulting in microbial death [25]. The antibacterial activity of SAEW-gel was found to be even more superior, with survival rates of *S. aureus* and *E. coli* dropping to 2.38% and 0.86%. This clearly illustrates that SAEW-gel exhibits significantly enhanced antibacterial properties compared to SAEW alone and conventional chitosan hydrogels.

The potent antibacterial activity of the SAEW-gel is intrinsically linked to the inherent antibacterial properties of SAEW. The antibacterial efficacy of SAEW has been demonstrated in studies [9,10,26], which attribute its potency to its ability to compromise the integrity of microbial membranes, disrupt membrane potential, and damage DNA. Additionally, it inhibits the enzymatic activities essential for microbial proliferation, thereby effectively eliminating bacteria. SAEW-gel dissolves almost entirely within 6 h, ensuring that SAEW is fully released through the proliferation mechanism, which is beneficial for the treatment of skin wound infections. SAEW-gel exhibits a very low water loss rate over 30 days and remains stable. This stability is crucial, as the gel contains a significant amount of SAEW. Its excellent water retention capabilities are pivotal in determining the mechanical properties of the hydrogel and the longevity of SAEW’s efficacy. The combination of good dissolution and long-term water retention creates a moist environment when the gel is applied to the skin’s surface, which is highly beneficial for wound healing and repair [27].

### 2.3. Stability of SAEW-Gel

**Figure 3:**
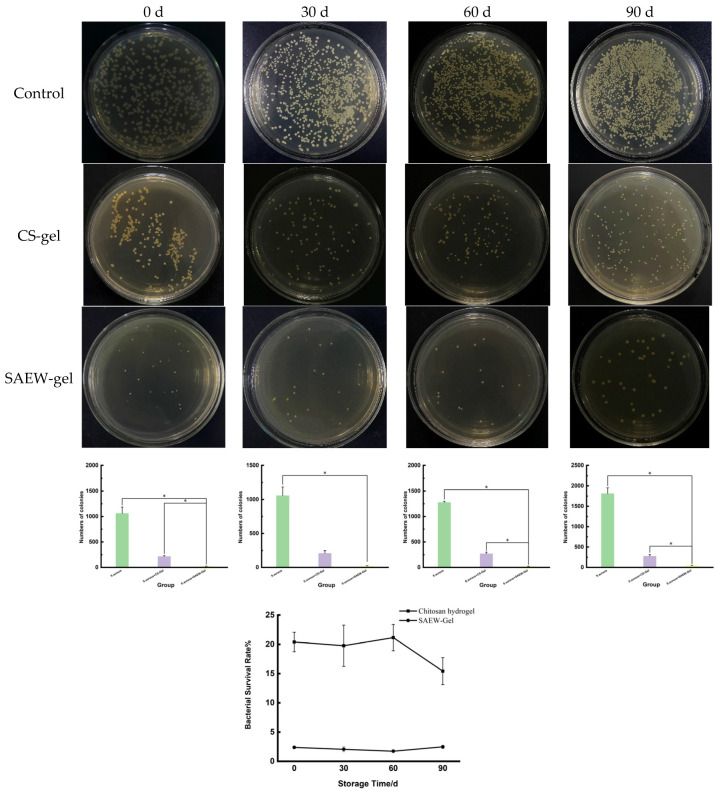
Antibacterial activity of hydrogel stored for different lengths against *S. aureus*. The asterisk (*) indicates a statistically significant difference (* *p* < 0.05).

Chitosan hydrogels and SAEW-gel were stored at 4 °C for 90 days. The survival rate of *S. aureus* treated with chitosan hydrogel ranged from 15.43% to 21.15%. In contrast, after treatment with SAEW-gel, the survival rate of *S. aureus* remained consistently low, at 1.74% to 2.48% (Figure 3). Overall, SAEW-gel exhibited superior antibacterial activity compared to chitosan hydrogel throughout the 90-day storage period, with no significant change in its antibacterial efficacy. These results indicate that SAEW-gel can effectively extend the service life and enhance the stability of SAEW.

### 2.4. Evaluation of Promoting Healing Effect on Skin Wounds

**Figure 4:**
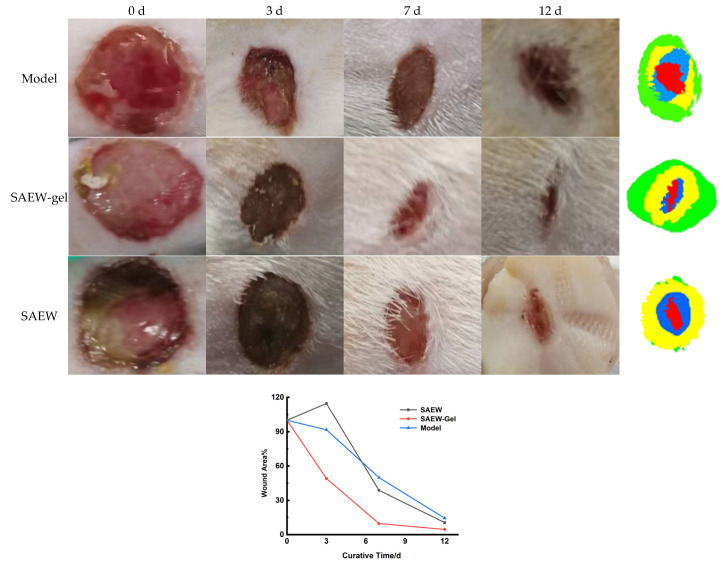
Skin wound healing in rats.

By the 12th day of treatment, 85.55% of the rats in the model group had healed, as had 89.36% in the SAEW group and a remarkable 95.41% in the SAEW-gel group (Figure 4). The SAEW-gel group exhibited the highest wound healing rate, surpassing the other two groups within the same 12-day treatment period, indicating its significant benefit in promoting rat skin wound repair.

### 2.5. Pathological Observation of Skin Wounds

**Figure 5:**
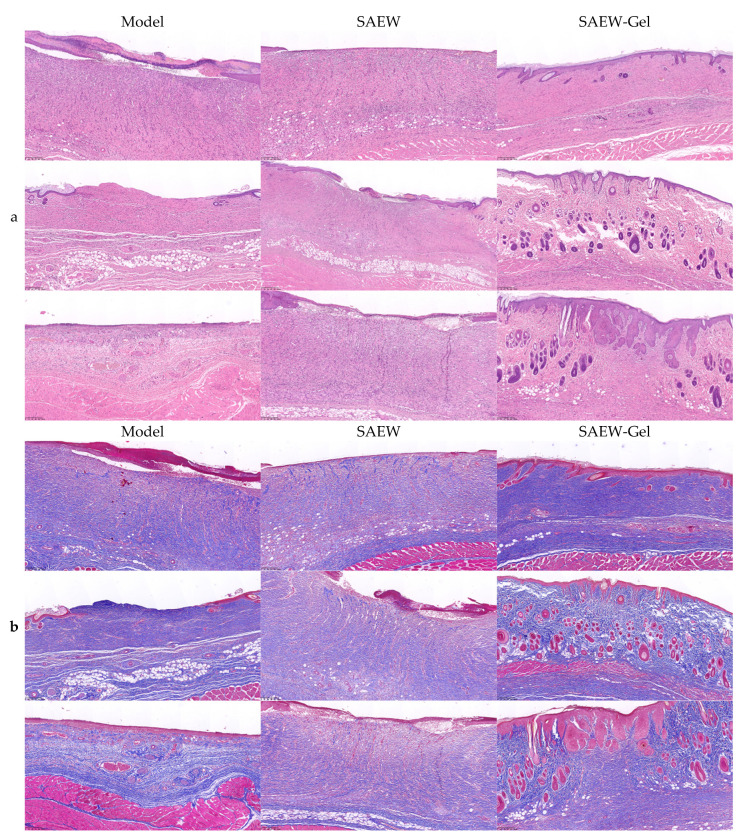
HE staining and Masson staining of rat skin wounds. (**a**) HE staining. (**b**) Masson staining. Microscope’s magnification is (100×).

In the model group, most skin lesions lacked squamous epithelial coverage, exhibited increased neutrophil presence on the surface, and displayed only minimal granulation tissue in the dermis. Some rats exhibited typical ulcerative changes in the center of the lesions, accompanied by more necrotic exudates on the surface. In contrast, the skin wounds of rats treated with SAEW showed signs of repair, with the injured area predominantly covered by thin squamous epithelium, granulation tissue in the dermis, and increased neutrophil infiltration at the wound bottom. A few rats exhibited hemorrhage and edema in the vicinity of the lower basal layer of the skin lesion area. In the SAEW-gel group, the rats’ skin was well-repaired, with the lesion area covered by proliferative squamous epithelium, lacking obvious granulation tissue, and primarily composed of collagen fibers and skin appendages (Figure 5a). The dermal collagen fiber content in the model group, SAEW group, and SAEW-gel group was notably increased. However, compared to the other two groups, the collagen deposition in the SAEW-gel group was more orderly, with thicker collagen fibers, denser accumulation, clearer lines, and higher maturity (Figure 5b).

### 2.6. Detection of Bacterial Load in Skin Wound

**Figure 6:**
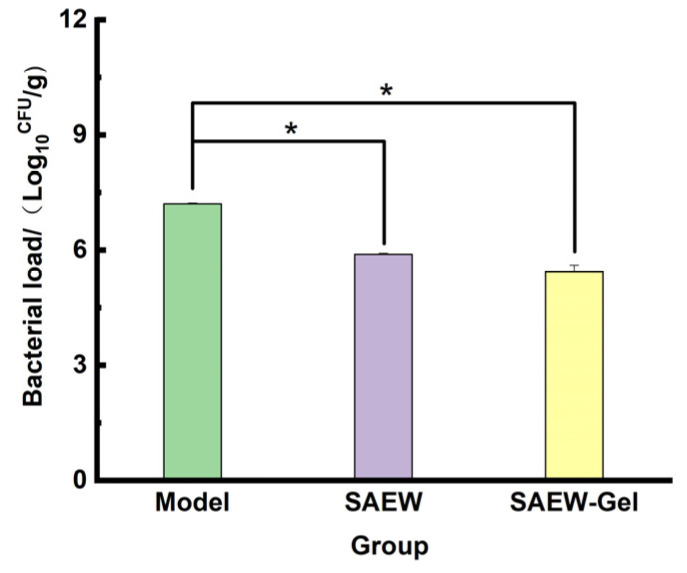
Bacterial load on rat wounds. The asterisk (*) indicates a statistically significant difference (* *p* < 0.05).

By the seventh day of treatment, the bacterial load on the skin wounds of the rats in the SAEW group and SAEW-gel group was significantly reduced compared to that in the model group (Figure 6), suggesting that SAEW-gel maintained its antibacterial efficacy on the rat skin surface.

### 2.7. Determination of Inflammatory Factors, Anti-Inflammatory Factors, and Collagen in Skin Wound Tissue

**Figure 7:**
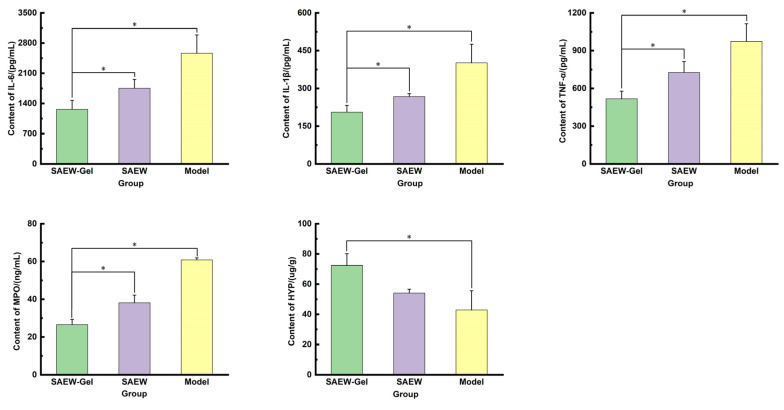
The analysis of ELISA. The asterisk (*) indicates a statistically significant difference (* *p* < 0.05).

The levels of IL-6, IL-1β, TNF-α, and MPO in the skin tissue of rats in the SAEW-gel group were notably lower than those in the model group and SAEW group (Figure 7), indicating that SAEW gel effectively inhibited the expression of inflammatory factor IL-6, reduced MPO content, and helped to eliminate inflammation. Additionally, the content of HYP in the skin tissue of rats in the SAEW-gel group was higher than that in the model group and SAEW group, suggesting that SAEW gel accelerates collagen tissue metabolism and fibrosis, thereby facilitating skin wound healing.

Wound healing is a highly orchestrated biological process, encompassing three distinct yet interconnected stages: hemostasis and inflammation, cell proliferation, and tissue remodeling. Following skin injury, blood vessels constrict, and platelets rapidly aggregate, triggering the clotting system to produce thrombin, which aids in clot formation to stop bleeding. Neutrophils arriving at the wound site proliferate to combat infection and are engulfed by macrophages [28]. These macrophages release growth factors, cytokines, and angiogenic factors at the injury site, fostering granulation tissue formation and angiogenesis, while also secreting pro-inflammatory factors such as TNF-α, IL-1β, and IL-6 [29]. During the initial stage of wound healing, the inflammatory phase, various cells within the skin tissue interact with inflammatory cells, such as through macrophage infiltration, lymphocyte aggregation, and neutrophil migration, to facilitate the clearance of damaging factors and the resolution of inflammation [30]. However, when the wound healing process is disrupted, inflammation can disrupt normal tissue function, leading to pathological fibrosis and scar formation.

Some studies [31] have shown that high expressions of TNF-α can induce the apoptosis of fibroblasts, hindering wound healing. Sustained high concentrations of IL-1β and IL-6 can lead to extracellular matrix degradation, exacerbate tissue injury, and delay wound healing by promoting wound infection and inflammation. MPO, a lysosomal protein stored in neutrophils, is closely linked to the number and activity of neutrophils and can serve as an important marker of inflammatory cells. The significantly lower levels of TNF-α, IL-1β, IL-6, and MPO in the skin wounds treated with SAEW-gel compared to other groups indicate that the inflammatory reaction was rapidly cleared and the damaged tissue was effectively repaired. During the wound healing process, the production of collagen by fibroblasts significantly influences the rate of wound closure [32]. HYP, a distinctive amino acid found in collagen, plays a crucial role in reflecting the metabolism and fibrosis of collagen tissue. The levels of HYP in the skin wounds treated with SAEW did not significantly differ from those in the model group. However, the HYP content in the skin wounds treated with SAEW-gel was significantly higher than in other groups, suggesting superior wound healing. When combined with the results of Masson staining, it is suggested that SAEW effectively eliminates bacteria in the treatment of rat skin wounds but does not notably affect collagen metabolism and fibrosis. In contrast, SAEW-gel, with its chitosan hydrogel component, promotes collagen metabolism and fibrosis while eradicating bacteria, a beneficial effect primarily attributed to the excellent biocompatibility of chitosan hydrogel.

## 3. Materials and Methods

### 3.1. Materials

This study utilized hydrochloric acid (Xilong Chemical Co., Ltd., Chengdu, China), sodium thiosulfate (Xilong Chemical Co., Ltd., Chengdu, China), sulfuric acid (Chengdu Cologne Chemical Co., Ltd., Chengdu, China), chitosan (>95%, Nantong Xingcheng Biological products Factory, Nantong, China), β-glycerol disodium phosphate hydrate (Shanghai McLean biochemical Technology Co., Ltd., Shanghai, China), and *E. coli* ATCC25922 and *S. aureus* ATCC25923 (gifts from the Veterinary Drug Safety Evaluation Laboratory of Sichuan Agricultural University).

### 3.2. Preparation of SAEW

The process of generating slightly acidic electrolyzed water involves adding a suitable quantity of a low-concentration dilute hydrochloric acid and sodium chloride aqueous solution to an electrolytic cell, which may or may not be equipped with an ion exchange membrane. During electrolysis, chlorine gas and H^+^ ions are produced at the anode. The dissolved H^+^ ions acidify the water, resulting in a pH range of 5.0 to 6.5. The chlorine gas reacts with water to create hydrochloric acid and hypochlorous acid (HClO). Simultaneously, at the cathode, only hydrogen gas is generated [33]. SAEW was prepared using an electrolytic water generator (model FX-SWS100, Yantai Fangxin Water Treatment Equipment Co., Ltd., Yantai, China). The process involved adding 5% hydrochloric acid and setting the water flow rate to 80 L/h. The electrolysis voltage of the instrument was adjusted to 5 V, with acid feeding at a rate of 30 s per cycle. After 30 min of electrolysis, SAEW with an available chlorine concentration (ACC) of 50 μg/mL was obtained.

### 3.3. Stability Investigation of SAEW

The prepared SAEW was put into glass bottles under the conditions of open with light, open without light, sealed with light, and sealed without light. The stability of the SAEW was characterized by the pH value, ORP value, ACC value, and antibacterial activity every 24 h.

### 3.4. Preparation of SAEW-Gel

The concentration of glacial acetic acid was diluted to 0.1mol/L by preparing SAEW. Then, an appropriate amount of chitosan was added and thoroughly mixed to obtain a 3% chitosan solution. Subsequently, β-glycerol disodium phosphate hydrate was dissolved in the SAEW. A mixture was meticulously prepared by stirring a 3% chitosan solution and 60% β-disodium glycerophosphate hydrate together in an ice bath, maintaining a volume ratio of 1:1. SAEW-gel was formed by incubating this mixture for 1 min at 37 °C. For comparison, a chitosan hydrogel was also prepared by substituting an equal volume of distilled water for the SAEW in the same process.

### 3.5. Appearance and SEM Observation of Gel

After gelation, the SAEW-gel was tilted or inverted for 5 min, and its morphological changes were visually inspected. For further examination, the freeze-dried gel was mounted on a sample stage and subjected to gold sputter coating. Its surface morphology was then analyzed using a scanning electron microscope. The SEM images were acquired using a Sigma 300 (Carl Zeiss, Oberkochen, Germany).

### 3.6. Antibacterial Effect In Vitro of SAEW-Gel

To assess the antibacterial activity of the SAEW, 200 μL of *S. aureus* or *E. coli* suspensions, both at a concentration of 1.5 × 10^8^ CFU/mL, were combined with 800 μL of SAEW and incubated for 1 min. Following this, 1 mL of a 1% sodium thiosulfate solution was added to neutralize the SAEW, and the mixture was allowed to stand for an additional 10 min. The bacterial solution was then diluted 10^3^-fold to prepare an appropriate mixture for subsequent use. A volume of 20 μL of this diluted mixture was then spread evenly onto a solid culture medium. The plates were incubated at 37 °C in a constant-temperature incubator for 24 h. The bacteriostatic efficacy of the SAEW was quantified using the plate counting method.

The hydrogel was placed at the bottom of a 48-well plate and incubated at 37 °C. Subsequently, 100 μL of *S. aureus* or *E. coli* suspension, with a concentration of 1.5 × 10^8^ CFU/mL, was applied to the surface of the hydrogel. The plate was then incubated at 37 °C for 2 h. Following this, the treated bacterial suspension was re-suspended in 900 μL of sterile PBS solution and diluted 10^3^-fold. The diluted bacterial solution was mixed thoroughly and coated on a solid medium. After incubating at 37 °C in a constant-temperature incubator for 24 h, the antimicrobial activity of the hydrogel was assessed using the plate counting method.

### 3.7. Swelling Test

SAEW-gel with an initial mass of M_0_ was weighed and placed into a centrifuge tube. Then, 5 mL of pH 7.4 phosphoric acid buffer was added, and the tube was placed in a water bath shaker (set at 37 ± 1 °C with a speed of 100 ± 5 r/min) for oscillation. At regular intervals, a set of samples was removed, the buffer was carefully decanted, and any residual liquid on the surface of the gel was gently blotted with filter paper. The wet weight of the gel was recorded at each time point. This procedure was repeated three times for accuracy. Using the initial wet weight of the gel as a reference, the percentage of the gel’s wet weight was calculated at different time points. The formula is as follows:

Wet weight percentage (%) = W_t_/W_0_ × 100% (1)

where W_t_ and W_0_ represent the time-dependent weight and initial (t = 0 min) weight of the hydrogels.

### 3.8. Water Retention Test

To evaluate the long-term water retention capability of SAEW-gel, gel with an initial mass of M_0_ was placed in a 37 °C incubator. The gel was then removed at predetermined intervals (12 h, 1 day, 2 days, 5 days, 10 days, 20 days, and 30 days) and its mass (M_n_) was weighed at each time point. The following formula was used to calculate the water loss rate (W) of the hydrogel:

*W* = (M_0_ − M_n_)/M_0_ (2)

### 3.9. Stability Investigation of SAEW-Gel

Changes in ACC value primarily indicate the stability of SAEW. However, the ACC value cannot be directly measured once the hydrogel is formed. A challenge in determining the ACC value of a hydrogel using an in vitro release method is that during the release process, a portion of the available chlorine in the SAEW may decompose, leading to inaccuracies in the detection data. Since the ACC value directly influences the antibacterial activity of SAEW, an alternative approach to reflect its changes is to use the in vitro antibacterial activity of the hydrogel over a 90-day storage period as an indirect indicator of the stability of SAEW within the hydrogel.

The prepared hydrogel was packed into a glass bottle and sealed at 4 °C. Samples were taken on the 0th, 30th, 60th, and 90th days. The hydrogel was then added to the bottom of a 48-well plate and incubated at 37 °C for 1 min. Following this, 100 μL of a 1.5 × 10^8^ CFU/mL *S. aureus* suspension was applied to the surface of the hydrogel, and the plate was incubated at 37 °C for 2 h. After re-suspending the treated bacterial suspension with 900 μL of sterile PBS solution, the mixture was diluted 10^3^-fold. The diluted solution was shaken thoroughly and evenly spread onto solid culture medium. After incubating at 37 °C in a constant-temperature incubator for 24 h, the bactericidal activity of the hydrogel was assessed using the plate counting method.

### 3.10. Evaluation of Promoting Healing Effect on Skin Wounds

Establishment of skin wound model: 60 SPF rats were anesthetized by intraperitoneal injection of 10% (*w*/*v*) chloral hydrate (0.3 mL/100 g). The hair on the left side of the spine was then shaved using a razor to expose the skin, followed by the application of hair removal cream to the exposed area. After a 10 min interval, the hair removal cream was gently removed with gauze soaked in warm water, ensuring the complete exposure of the rat’s skin. The exposed skin was lifted, and a sterilized 1 cm diameter corneal ring punch was used to create a round wound of the same diameter. The wound was disinfected, and a 50 μL suspension of *S. aureus* (1 × 10^7^ CFU/mL) was evenly applied to the wound site. As the *S. aureus* suspension penetrated the damaged skin, a film was applied over the wound surface. The wound was then covered with disinfectant gauze and secured with medical tape. Two days later, the membrane covering the wound was removed, and the disinfectant gauze and medical tape were also removed. The presence of yellow pus in the wound indicated the successful establishment of a rat skin wound model with a diameter of 1 cm.

Group treatment of skin wounds: Following the successful establishment of the skin wound model, the rats were randomly allocated into three groups, each comprising 20 rats (*n* = 20). These groups were designated as the model group, the SAEW-gel group, and the SAEW group. The treatment protocols for each group were as follows: Rats in the model group received no treatment. In the SAEW-gel group, 2 g of SAEW-gel was applied to the skin wound to form an in situ hydrogel, which was administered twice daily. Before each application of the SAEW-gel precursor solution, any remaining hydrogel was carefully removed. For the SAEW group, the rats’ skin wounds were directly treated with an appropriate amount of SAEW twice daily. Following treatment, the rats’ wounds were dressed with gauze and medical bandages.

On days 0, 3, 7, and 12 of treatment, the rats were anesthetized with 10% chloral hydrate solution (0.3 mL/100 g). The gauze and medical bandages covering the wounds were then removed, and the wounds were photographed. Using ImageJ software (version 1.53t), the wound area was selected and measured. The wound closure rate was calculated as (initial wound area − current wound area)/initial wound area × 100%.

Pathological observation of skin wound: On the 12th day of treatment, six rats from each group were randomly selected. The skin tissue around the wound was carefully excised using ophthalmic scissors and placed in normal saline to clean off any blood on the surface. Excess saline was gently wiped away with gauze, and the samples were then fixed with 4% (*w*/*v*) paraformaldehyde. Hematoxylin and eosin staining was employed to assess the growth of granulation tissue and the maturation of skin, while Masson trichrome staining was utilized to evaluate collagen deposition.

Detection of bacterial load in skin wound: On the 7th day of treatment, three rats from each group were randomly selected for anesthesia. Aseptically, a corneal ring drill was used to extract tissue samples from the central and marginal areas of the wound. The samples were minced and mixed with PBS at a ratio of 1:9 by mass. This mixture was then added to a glass homogenizer for thorough homogenization. A 1 mL aliquot of the homogenate was diluted 10^3^-fold, and 50 μL of this dilution was evenly spread onto a solid culture medium. The plates were incubated in a 37 °C constant-temperature incubator for 24 h. The bacterial count was determined using the plate counting method. The formula for calculating bacterial load is as follows:

*Bacterial load (CFU/g)* = colony count (CFU) * dilution multiple/tissue weight (g) (3)

Determination of inflammatory factors, anti-inflammatory factors, and collagen in skin wound tissue: Six rats from each group were randomly selected and euthanized on the 12th day of treatment. The skin tissue surrounding the wound was carefully excised using ophthalmic scissors and placed in a saline solution to remove any blood from the skin’s surface. Excess saline was gently wiped off, and the tissue was then weighed and cut into chunks. The chopped tissue was mixed with PBS at a ratio of 1:9 by mass and added to a glass homogenizer. The tissue was then homogenized completely on ice to ensure thorough disruption. The crushed tissue homogenate was further broken down by ultrasonic waves. Finally, the homogenate supernatant was centrifuged at 4 °C at 5000 rpm. The resulting supernatant was used for the detection of interleukin-6 (IL-6), interleukin-1β (IL-1β), tumor necrosis factor-α (TNF-α), myeloperoxidase (MPO), and hydroxyproline (HYP) levels, following the specific instructions provided with the respective kits.

## 4. Conclusions

We successfully developed a novel SAEW-based hydrogel (SAEW-gel) by combining SAEW with chitosan and β-glycerol disodium phosphate. Compared to SAEW alone, SAEW-gel demonstrated significantly improved physicochemical properties, including enhanced water retention and long-term stability, effectively mitigating chlorine loss over time. In vitro experiments confirmed that SAEW-gel exhibited superior antibacterial activity against *Staphylococcus aureus* and *Escherichia coli*, with a lower bacterial survival rate than SAEW or chitosan hydrogel alone. In vivo wound healing studies further demonstrated that SAEW-gel promoted faster wound closure, enhanced collagen deposition, and reduced inflammatory responses more effectively than SAEW, achieving a 95.41% healing rate by day 12. These findings suggest that SAEW-gel holds strong potential as a multifunctional wound dressing, offering improved stability, broad-spectrum antimicrobial effects, and accelerated healing. In future work, we plan to evaluate its performance in chronic wound models and compare it with standard clinical treatments to further validate its translational potential.

## Data Availability

The original contributions presented in the study are included in the article. Further inquiries can be directed to the corresponding author.
